# *In Vivo* Lipid Regulation Mechanism of Polygoni Multiflori Radix in High-Fat Diet Fed Rats

**DOI:** 10.1155/2014/642058

**Published:** 2014-04-30

**Authors:** Pei Lin, Yan Ran He, Jian Mei Lu, Na Li, Wan Gen Wang, Wen Gu, Jie Yu, Rong Hua Zhao

**Affiliations:** Yunnan University of Traditional Chinese Medicine, Kunming, Yunnan 650500, China

## Abstract

Mechanisms of the water extracts of Polygoni Multiflori Radix (PMR) and its processed products (PMRP) on liver lipid metabolism were observed in this paper. Aqueous extract of PMR and PMRP was given to nonalcoholic fatty liver model rats, respectively. PMR was better in reducing the contents of very low density lipoprotein (VLDL) than PMRP and the positive control groups. In the aspect of regulating TG, medium dose PMR reduced the activity of diacylglycerol acyltransferase (DGAT) to 1536 ± 47.69 pg/mL (*P* < 0.001) and promoted the expression of hepatic lipase (HL) to 23.59 ± 0.2758 U/mL (*P* < 0.05). HL promotion ability of medium dose PMR was similar with the simvastatin positive control. Both medium and high dose of PMR showed significant alterations in TC, which were related to the downregulation effects on hydroxyl methyl-glutaryl coenzyme A reductase (HMGCR) and upregulation effects on cholesterol 7-alpha-hydroxylase or cytochrome P450 7A (CYP7A). Quantitative relationships research indicated that the prominent effect on inhibiting the content of HMGCR (*r* = 0.756, *P* < 0.05) was strongly positive correlated with to the TC regulation effects. Effects of PMR on enhancing decomposition rate or reducing *de novo* synthesis rate of TG and TC were better than PMRP.

## 1. Introduction

Fatty liver disease (FLD), a kind of lipid metabolic disorder of liver, is a reversible condition in which large vacuoles of triglyceride fat accumulate in liver cells via the process of steatosis (abnormal retention of lipids within a cell). According to the different inducements of fatty liver, FLD is divided into alcoholic fatty liver disease (AFLD) and nonalcoholic fatty liver disease (NAFLD). NAFLD is increasingly recognized as the hepatic manifestation of insulin resistance and the systemic complex known as metabolic syndrome [1, 2]. NAFLD is the most common form of chronic liver disease in adults in the United States, Australia, Asia, and Europe [3–5]. NAFLD is also gaining recognition as a significant early sign of liver cirrhosis and liver cancer [6]; prevalence estimates of NAFLD have used a variety of laboratory and imaging assessments.

In NAFLD pathogenic process, the accumulation of lipid within the liver, especially total cholesterol (TC) and triglycerides (TG) accumulation, has been confirmed by dynamics research. These researches point out that TC, TG, and esterification of free fatty acid (FFA) accumulation in the liver for the treatment of NAFLD might have direct influence [7, 8].

**Figure 1:**
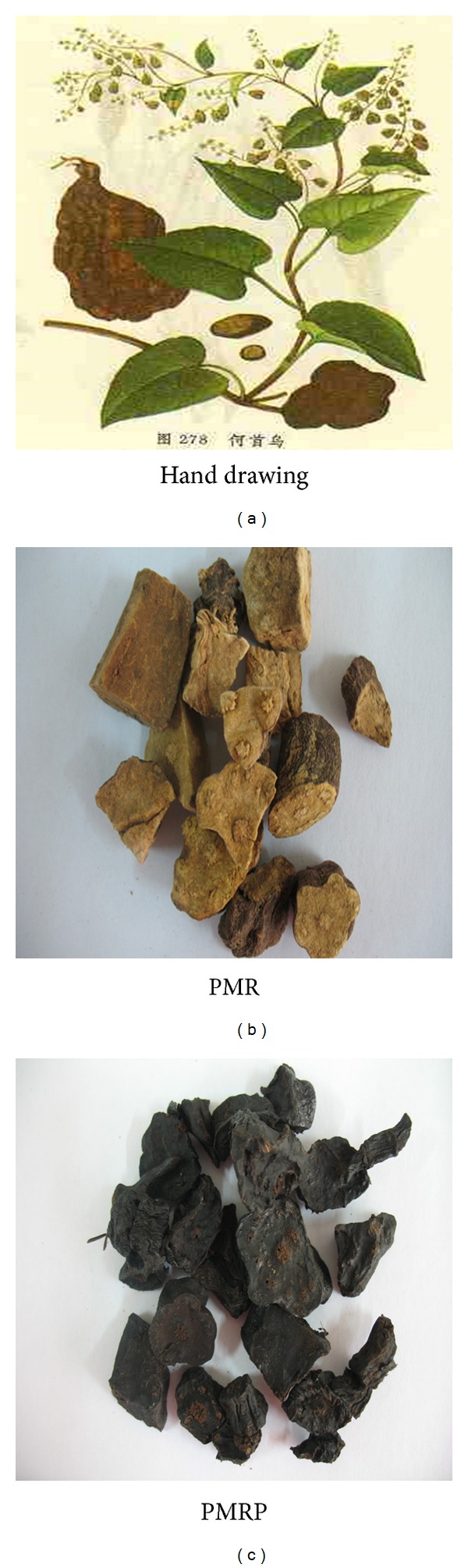
Photographs of Polygoni Multiflori Radix and its processed products.

Polygoni Multiflori Radix (PMR, Heshouwu in Chinese) and Polygoni Multiflori Radix Praeparata (PMRP, Zhiheshouwu in Chinese) are originated from the root of* Polygonum multiflorum* Thunb. (Polygonaceae) (Figure 1). They mainly contain anthraquinone, stilbene glycosides, phospholipids, and other ingredients and are used in the prevention and treatment of NAFLD, hyperlipidemia, or related diseases in oriental counties for centuries [9].

**Figure 2:**
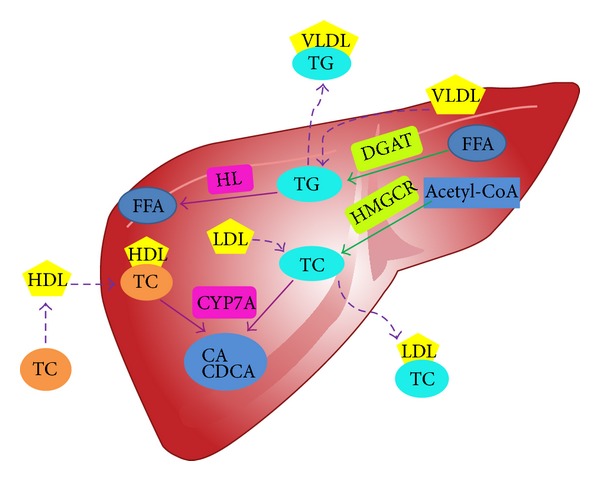
Lipid synthesis and lipolysis procedure of TG and TC.

Previously, a sensitive, accurate, and rapid* in vitro* model (the steatosis L02 cells, obtained after being cultured with 1% fat emulsion, 10% fetal bovine serum (FBS), and RPMI 1640 medium for 48 h) was used in our research group to investigate the lipid regulation effects of 2, 3, 5, 4′-tetrahydroxystilbene-2-O-*β*-D-glucoside (TSG), emdin, and physcion [10]. Hereafter,* in vivo* model (high-fat diet fed rats) was applied to explore the lipid regulation effects of extracts of PMR and PMRP [11]. The results show that both the raw crude drug and its processed products have significant lipid-lowering activities; however, obvious target organ selectivity was found. PMR was considered to possess better effects in lipid regulation in liver sample and was recommended for the treatment of early stage NAFLD. On the other hand, PMRP displayed better effects in lipid regulation in blood circulation system for the treatment of hyperlipidemia. However, the lipid regulation mechanism of* P. multiflorum* is still not clearly elucidated. So we chose the following four key enzymes as the investigation objectives, in this paper (Figure 2).

3-Hydroxy-3-methylglutaryl-CoA reductase (HMGCR), most abundantly expressed in the liver, plays a central role in the regulation of TC concentration. HMGCR is a key enzyme catalyzing cholesterol in* de novo* synthesis pathway* in vivo*. HMGCR activity directly affects the speed of cholesterol synthesis and the level of cholesterol [12]. Clinical results confirm that HMGCR inhibitor reduced plasma concentrations of TC and TG, low density lipoprotein (LDL), and very low density lipoprotein (VLDL) and increased plasma concentrations of high density lipoprotein (HDL). Therefore, the inhibition of this enzyme could contribute to reduction of synthesis of cholesterol.

Cholesterol 7a-hydroxylase (CYP7A) is the first and rate-limiting enzyme in bile acid synthesis pathway and is expressed only in the liver. Lack of CYP7A results in high levels of plasma cholesterol, whereas induction of CYP7A prevents elevation of blood cholesterol in rodents fed by a cholesterol-rich diet indicating its importance in maintaining plasma cholesterol homeostasis. CYP7A is tightly regulated by feedforward of cholesterol and negative feedback of bile acids [13].

The diacylglycerol acyltransferase (DGAT) is rate-limiting enzyme for triglyceride synthesis. DGAT catalyzes the final step in TG biosynthesis by converting diacylglycerol (DAG) and fatty acyl-coenzyme A into TG [14].

Hepatic lipase (HL) is a lipolytic enzyme that contributes to the regulation of TG levels. Hepatic lipase facilitates the clearance of TG from VLDL pool, and this function is governed by the composition and quality of HDL particles. HDL regulates the release of HL from the liver and HDL structure controls HL transport and activation in the circulation [15]. HL could catalyze the chylomicrons (CM) and promote the hydrolysis of triglycerides in VLDL.

Based on the key role of above enzyme in the lipid metabolism, we used the* in vivo* model (high-fat diet fed rats) to investigate lipid regulation mechanisms and possible regulatory targets of TC and TG by PMR and PMRP further and systematically.

## 2. Materials and Methods

### 2.1. Samples [11]

PMR was collected in Luquan, Yunnan, by the authors. The plants were identified as the root of* Polygonum multiflorum* Thunb. by Prof. Rong-hua Zhao, Yunnan University of Traditional Chinese Medicine. PMRP was made by PMR with black bean decoction according to the method recorded in the Pharmacopoeia of People's Republic of China [8]. Processed products were detected by high liquid chromatography (HPLC); the content of stilbene glycoside was greater than 0.70%, in accordance with the Pharmacopoeia standards, while the content of stilbene glycoside of raw products was greater than 1.0%.

### 2.2. Preparation of Extraction of PMR and PMRP [11]

300 g powder of PMR and 472 g powder of PMRP were extracted for 1 hr with 10 times boiling water. Then the residue was extracted for 40 min with 10, 8, and 6 times volume boiling water, respectively. Extracts were combined, condensed, and lyophilized. The concentrations of PMR and PMRP extracts were 0.6980 g/mL and 0.8580 g/mL, respectively.

### 2.3. Animals Groups [11]

SD rats were provided by the Experimental Animal Center of Yunnan University of Traditional Chinese Medicine. They were aged 8 weeks and weighed 245 ± 20 g and were acclimated in the controlled environment (temperature 22 ± 1°C; 60 ± 10% humidity; and a 12 h/12 h light/dark cycle) with free access to water and a commercial laboratory complete food. All animal experiments were performed in compliance with the Animal Experimental Ethics Committee of Yunnan University of Traditional Chinese Medicine. All reasonable efforts were made to minimize the animals' suffering.

120 SD male rats were randomly divided into 10 groups (Table 1): normal control group (A), model group (B), water extraction group of PMR (low, medium, and high dose groups: C, D, and E), water extraction group of PMRP (low, medium, and high dose groups: F, G, and H), and positive control groups (fenofibrate and simvastatin control: I and J). In addition to the normal control group, other groups were fed with a high-fat diet (containing 1% cholesterol, 10% lard, 0.2% methyl thiouracil, and 88.8% usual feed) to the end of the experiment (42 days).

### 2.4. Drug Delivery Process after the Success of Modeling [11]

After giving high-fat diet for 18 days, group C to H received the PMR and PMRP treatments till 42 days, the end of the research. In the meantime, normal control group and hyperlipidemia model group were given 0.9% saline 1 mL. Positive groups I and J received 0.033 g·kg^−1^ fenofibrate and 0.0012 g·kg^−1^ simvastatin daily, respectively. The low dose group of PMR and PMRP was given 0.405 g·kg^−1^ and 0.810 g·kg^−1^ daily [15]; the middle and high dosages of PMR and PMRP were 2 and 4 times of the low dosages, respectively. All rats were fasted for 2 h every day before administration of therapeutic agents (Table 1).

### 2.5. The Preparation and Detection of Animal Liver Homogenate

**Figure 3:**
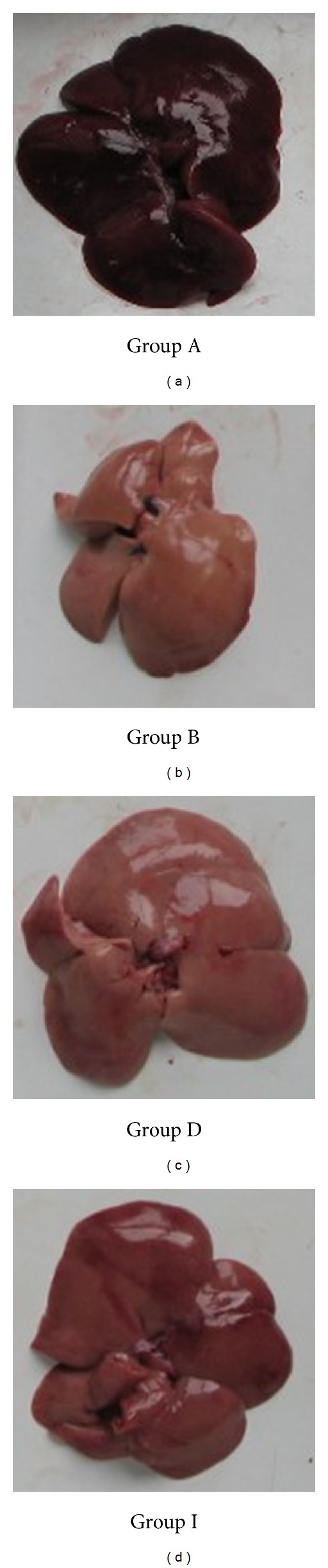
Liver tissue samples. Group A normal control group. Group B model group. Group D water extraction group of PMR (medium dose). Group I positive control group (simvastatin).

Rats were sacrificed by cervical dislocation. Liver samples were collected (Figure 3) and weighed after washing with 0.9% saline. 100 mg tissues were rinsed with PBS and homogenized in 1 mL of PBS and then stored overnight at −20°C. After two freeze-thaw cycles were performed to break all cell membranes, the homogenates were centrifuged for 5 minutes at 5000 g, 2–8°C. The supernatants were collected and analysed immediately.

Contents of TC, TG, LDL, and HDL in the supernatant were measured by AB-1020 automatic biochemical analyzer (Sunostik Medical Technology Co., Ltd.) and assay kits purchased from Shanghai Rongsheng Biological Pharmaceutical Co., Ltd., and Sichuan Maker Biotechnology Co., Ltd., China.

The VLDL, DGAT, HMGCR, HL, and CYP7A contents were tested by assay kits purchased from Cusabio Biotech Co., Ltd., China.

### 2.6. Statistical Analysis

All data in this research were expressed in the form of mean ± SD. All data were analyzed by single factor analysis of variance (ANOVA) statistics and the test results of *P* < 0.05, *P* < 0.01, and  *P* < 0.001 as a statistically significant difference criterion.

Relationships between these enzymes and proteins and TG and TC were assessed with Pearson's correlation coefficient. Results were classified into two significance levels using the *P* value of 0.05.

## 3. Results

### 3.1. Effects of Lipid Regulation Using Raw and Processed Radix Polygoni Multiflori [11]

Morphologic observations were carried in every group. Livers in high-diet fed group were obviously smaller and paler than normal livers. Treatment of simvastatin, PMR, and PMRP relieved the steatosis procedure; however, none of these treatments could reverse it (Figure 3).

As listed in Table 2, TC, TG, and LDL-C in liver tissue were all significantly higher than in model rats. Both PMR and PMRP revealed TC-lowering effects; however, dose-dependent TC- and TG-lowering effects were observed only in PMR groups.

### 3.2. Effects of PMR and PMRP on VLDL in Liver Samples

**Figure 4:**
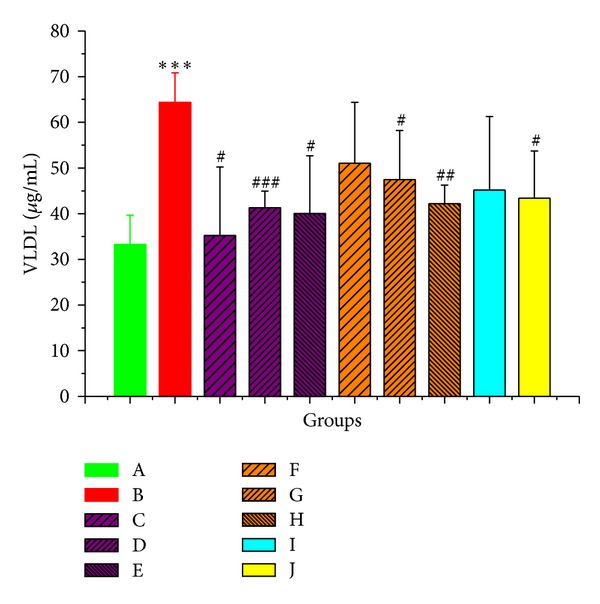
The content of VLDL of liver homogenate in all groups. The * indicates a significant difference compared with control group; *P < 0.05, **P < 0.01, and ***P < 0.001. The # indicates a significant difference compared with model group; ^#^ *P* < 0.05, ^##^ *P* < 0.01, and ^###^ *P* < 0.001.

42 days of high-fat diet intake significantly increased the liver VLDL level from 33.22 ± 6.445 ug/mL to 64.36 ± 6.455 ug/mL (*P* < 0.001) in rats. Fortunately, the liver VLDL levels were reduced in PMR, PMRP, and positive control drugs. From comparison of PMR groups and PMRP groups, PMR was better in reducing VLDL than PMRP. Low dosage of PMR could reduce the content of VLDL to 35.20 ± 15.03 ug/mL, which was similar to the normal group. VLDL-lowering effects were even better than the positive control groups (Figure 4).

### 3.3. Expression of Key Enzyme of Triglyceride (TG) Metabolism

The activities of DGAT and HL were investigated as the key enzymes of TG metabolism.

**Figure 5:**
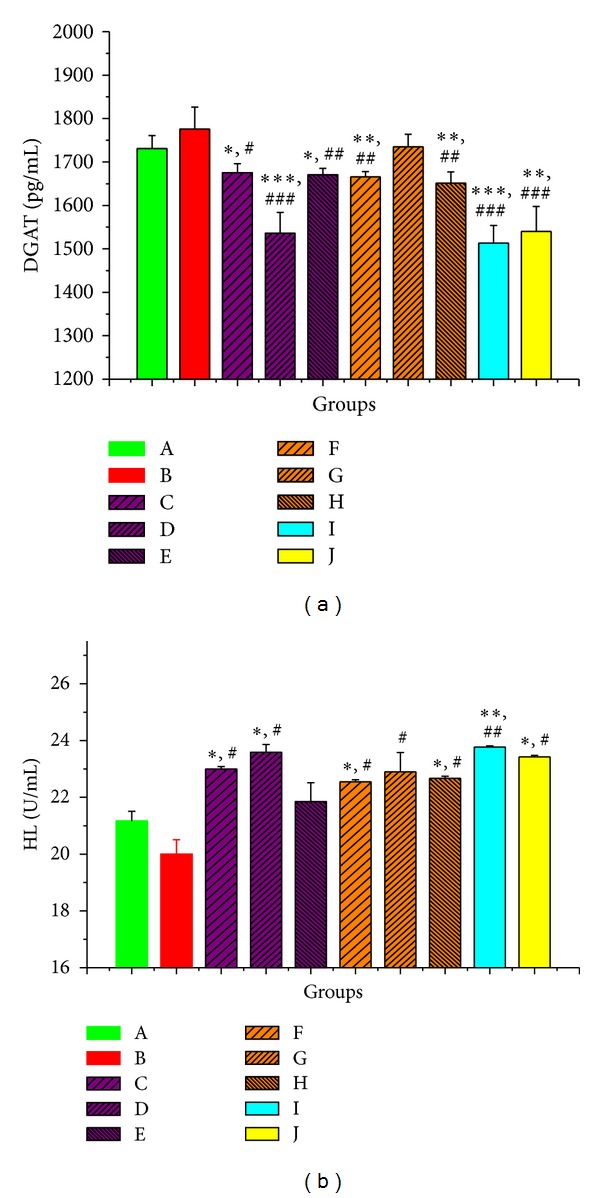
The activity of key enzymes of TG metabolism. The * indicates a significant difference compared with control group; *P < 0.05, **P < 0.01, and ***P < 0.001. The # indicates a significant difference compared with model group; ^#^ *P* < 0.05, ^##^ *P* < 0.01, and ^###^ *P* < 0.001.

Considering the two indexes in Figure 5, the effects of aqueous extract of PMR on enzyme activity were better than those of PMRP. Medium dose group of PMR showed more prominent effect; they reduced the activity of DGAT from 1776 ± 50.44 pg/mL to 1536 ± 47.69 pg/mL and promoted the expression of HL to 23.59 ± 0.2758 U/mL. HL expression regulation ability of PMR was even similar to the positive control group. The way of PMR to regulate the TG content in the liver was to reduce the activity of DGAT and promote the expression of HL. The regulations of DGAT were not very obvious in all PMRP groups, while the promotion effects of HL were much better.

### 3.4. Expression of Key Enzyme of Total Cholesterol (TC) Metabolism

**Figure 6:**
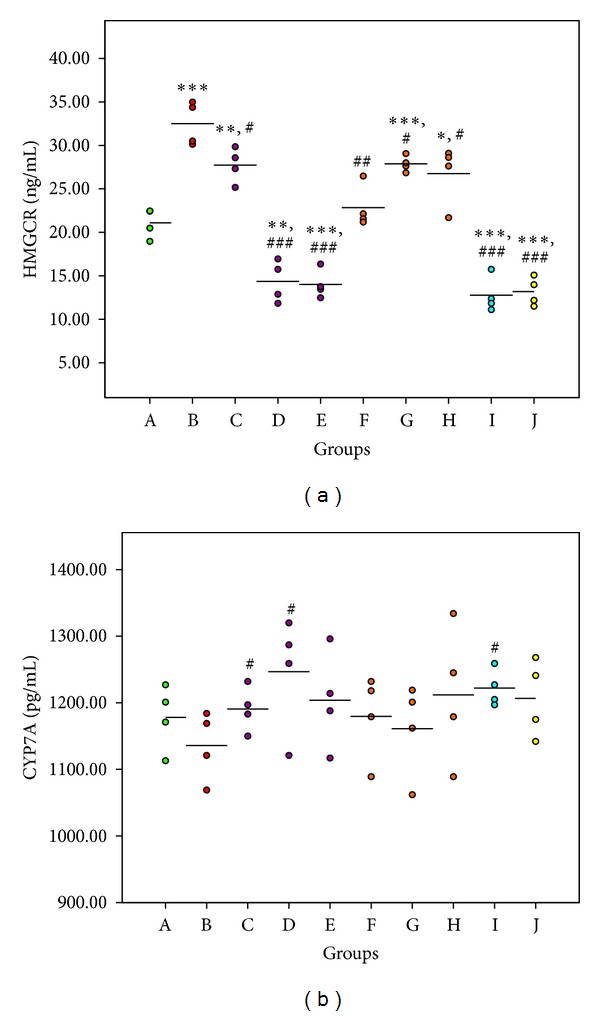
The activity of key enzymes of TC metabolism. The * indicates a significant difference compared with control group; *P < 0.05, **P < 0.01, and ***P < 0.001. The # indicates a significant difference compared with model group; ^#^ *P* < 0.05, ^##^ *P* < 0.01, and ^###^ *P* < 0.001.

TC level in high-fat diet group was significantly higher than that in the control group (Table 3); no TC melioration effect was observed in the low dosage PMR group; however, both the middle (group D) and high dosage (group E) PMR groups showed significant changes in TC regulation. These TC regulation effects were related to the downregulation on HMGCR and upregulation on CYP7A. Comparing the expression of these two key enzymes (Figure 6), HMGCR played a leading role in TC metabolism; the ability of middle and high dosage PMR was similar to the positive control group. In the meantime, all dosage groups of PMRP showed the TC melioration effect; however, these are not better than PMR.

### 3.5. Relationships between Enzymes and Proteins and TC and TG

Pearson's correlation coefficients between these enzymes and proteins and TG and TC were displayed in Table 4. HMGCR activities (*r* = 0.756, *P* < 0.05) were strongly positive correlated with TC. Moreover, LDL was positive correlated with TC, CYP7A was negative correlated with TC, and VLDL was positive correlated with TG. However, we did not find any significant relationship between DGAT and HL and TG regulation effects.

## 4. Discussion and Conclusion

Liver was the main organ responsible for lipid metabolism. Liver cells played important roles in lipid uptake, transport, metabolism, and excretion. Generally, the fat metabolism disorder leaded to liver steatosis which was the initial step of fatty liver disease. Fat metabolism related to fatty liver induced triglyceride metabolism, cholesterol metabolism, phospholipid metabolism, and priority to triglycerides metabolic disorder.

However, there was no specific drug for NAFLD treatment. Considering the effectiveness and acceptable prices of traditional Chinese medicine (TCM), the prevention and treatment of NAFLD and hyperlipidemia by TCM were a research hotspot. PMR and PMRP, which displayed great clinical effect in treating NAFLD, seemed to be potential choices.

Our research group had focused on the lipid regulation effect of PMR and PMRP for decades; we had confirmed the great lipid reducing effect of PMR and PMRP by both* in vitro* [10] and* in vivo* [11] assays. However, the lipid regulation mechanism of* P. multiflorum* was still not clearly elucidated. So in this research we chose four key enzymes, DGAT, HL, HMGCR, and CYP7A, as the major objectives, to seek the possible regulatory targets in the metabolisms of TC and TG by PMR and PMRP.

After giving high-fat diet for 42 days, we found that TC, TG, LDL, and VLDL in liver tissue were all significantly higher than in model rats. DGAT and HMGCR in model group were upregulated, and CYP7A and HL were all downregulated. These showed that the method of the rats induced into NAFLD was successful.

Compared with model group, PRM and PRMP had good lipid-lowering effects; they could not only enhance decomposition activity but also reduce synthase activity. TC and TG melioration effects of PRM were better. In particular, middle dose group of PMR showed more prominent effect on inhibiting the content of HMGCR and DGAT, which could block the synthesis of TC and TG, respectively. Key enzyme regulation ability of PMR was similar to the positive control group. At the same time, low dose group of PMR showed the best VLDL-reducing effect, and the VLDL-reducing effect of PMRP was in a dose-dependent manner.

According to the analysis of Pearson's correlation coefficients, HMGCR activity (*r* = 0.756, *P* < 0.05) was strongly positive correlated with TC regulation effects. This was consistent with the above results. In a conclusion, TC regulation of PMR was mediated by HMGCR. In some extension, PMR showed similar activities and mechanisms with statins. We also found the better TG regulation effects in some dosage of PMR and PMRP; however, there was no significantly relationship between DGAT, HL, VLDL, and TG. Therefore, we assumed that the regulation on TG might be related to some other mechanisms or targets.

Previous researches [16, 17] pointed out that water extract and total glycosides of PMR had shown a good lipid-lowering activity in animal experiments; they could reduce the contents of the TG and TC in rats caused by high-fat diet. Moreover, the effect of total glycosides on reducing the TG in hyperlipidemia rat lack of Apo E gene was even better than the positive drugs. These were in line with our results that high dosage of PMR had the similar TG-lowering effect with statins.

Other researches [18, 19] consider that the lipid-lowering activity of PMR was associated with the inhibition of HMGCR, the reduction of VLDL and LDL contents, and the decreasing of TG and TC absorption. In this paper, we reported the effects on the activity of DGAT, HL, and CYP7A for the first time. This research could contribute to our knowledge on the lipid regulation mechanism of PMR in high-fat diet fed rats.

TG content in the liver was affected not only by the amount directly absorbed from the food but also by the* de novo* synthesis by FFA [8, 20]. Therefore, whether FFA supply chain was affected by PMR and PMRP will be in great worthy of study in the future. On the other hand, previous literatures [21, 22] also showed that insulin resistance was often observed in the NAFLD patients. The increasing of insulin concentration could reduce the oxidation and decomposition rate of TG, so that TG content in the cells would increase [23]. Therefore, whether PMR and PMRP could control the insulin resistance status in NAFLD patients also will be a subject in our future research plan.

## Figures and Tables

**Table 1:** Animal grouping and treatments in this research.

Groups	Diets	Treatment (from the nineteenth day of the experiment)	Dosage (g/kg body weight)
A	Normal diets	Physiological saline	1 mL per rat
B	High-fat diets	Physiological saline	1 mL per rat
C	High-fat diets	Water extraction of PMR	0.4050
D	High-fat diets	Water extraction of PMR	0.8100
E	High-fat diets	Water extraction of PMR	1.620
F	High-fat diets	Water extraction of PMRP	0.8100
G	High-fat diets	Water extraction of PMRP	1.620
H	High-fat diets	Water extraction of PMRP	3.240
I	High-fat diets	Simvastatin	0.001200
J	High-fat diets	Fenofibrate	0.03300

**Table 2:** Lipid indexes in the liver samples.

Groups	TC (mg/dL)	TG (mg/dL)	LDL-C (mg/dL)
A	66.63 ± 4.093	147.22 ± 6.180	10.74 ± 2.186
B	100.2 ± 19.22***	200.0 ± 32.56***	28.36 ± 12.57**
C	105.8 ± 15.01***	179.8 ± 18.56***	28.79 ± 7.821***
D	87.71 ± 17.19**	180.8 ± 15.94***	24.49 ± 6.547***
E	57.18 ± 6.754^∗∗,###^	153.6 ± 27.34^#^	24.86 ± 4.385***
F	66.29 ± 28.08^#^	162.1 ± 39.88	38.39 ± 18.53***
G	89.48 ± 18.75**	205.8 ± 29.90***	48.98 ± 12.02^∗∗∗,#^
H	69.82 ± 24.30^#^	165.0 ± 32.11	41.89 ± 16.49***
I	43.67 ± 2.936^∗∗∗,###^	150.8 ± 18.82^##^	9.990 ± 3.548^##^
J	44.66 ± 5.379^∗∗∗,###^	195.8 ± 25.96***	15.79 ± 9.505

The (∗) indicates a significant difference compared with control group; *P < 0.05, **P < 0.01, and ***P < 0.001.

The (#) indicates a significant difference compared with model group; ^#^ *P* < 0.05, ^##^ *P* < 0.01, and ^###^ *P* < 0.001.

**Table 3:** Contents of TC, HMGCR, and CYP7A in every group.

	TC (mg/dL)	HMGCR (ng/mL)	CYP7A (pg/mL)
A	66.63 ± 4.093	21.09 ± 1.687	1178 ± 49.00
B	100.2 ± 19.22***	32.50 ± 2.533***	1110 ± 54.64
C	105.8 ± 15.01***	27.73 ± 1.994^∗∗,#^	1191 ± 33.96^#^
D	87.71 ± 17.19**	14.35 ± 2.389^∗∗,###^	1247 ± 87.46^#^
E	57.18 ± 6.754^∗∗,###^	14.01 ± 1.655^∗∗∗,###^	1203 ± 73.91
F	66.29 ± 28.08^#^	22.84 ± 2.464^##^	1180 ± 64.37
G	89.48 ± 18.75**	27.88 ± 0.920^∗∗∗,#^	1161 ± 70.16
H	69.82 ± 24.30^#^	26.76 ± 3.432^∗,#^	1212 ± 103.59
I	43.67 ± 2.936^∗∗∗,###^	12.77 ± 2.050^∗∗∗,###^	1222 ± 27.74^#^
J	44.66 ± 5.379^∗∗∗,###^	13.19 ± 1.640^∗∗∗,###^	1207 ± 58.09

The (∗) indicates a significant difference compared with control group; *P < 0.05, **P < 0.01, and ***P < 0.001.

The (#) indicates a significant difference compared with model group; ^#^ *P* < 0.05, ^##^ *P* < 0.01, and ^###^ *P* < 0.001.

**Table 4:** Relationships between enzymes and proteins and TG and TC.

	Correlation coefficient and significance	Pearson's correlation coefficient	Significance (*P*)
TC	LDL	0.487	0.153
	HMGCR	0.756	0.011
	CYP7A	−0.453	0.189

TG	VLDL	0.490	0.150
	DGAT	0.192	0.596
	HL	−0.019	0.958

## Abbreviations

CM: Chylomicrons
CYP7A: Cholesterol 7-alpha-hydroxylase or cytochrome P450 7A
DAG: Diacylglycerol
DGAT: Diacylglycerol acyltransferase
FBS: Fetal bovine serum
FFA: Free fatty acid
FLD: Fatty liver disease
HMGCR: 3-Hydroxy-3-methylglutaryl-CoA reductase
HDL: High density lipoprotein
HL: Hepatic lipase
HPLC: High liquid chromatography
LDL: Low density lipoprotein
NAFLD: Nonalcoholic fatty liver disease
PMR: Polygoni Multiflori Radix
PMRP: Polygoni Multiflori Radix Praeparata
RPMI 1640: Roswell Park Memorial Institute medium 1640
TC: Total cholesterol
TCM: Traditional Chinese medicine
TG: Triglyceride
TSG: 2, 3, 5, 4′-Tetrahydroxystilbene-2-O-*β*-D-glucoside
VLDL: Very low density lipoprotein.
